# Discovery and Characteristics of a Novel Antitumor Cyclopeptide Derived from Shark

**DOI:** 10.3390/bioengineering10060674

**Published:** 2023-06-01

**Authors:** Fu Li, Minghua Lei, Junye Xie, Shujun Guo, Weicai Li, Xiujuan Ren, Teng Wang, Songxiong Lin, Qiuling Xie, Xiaojia Chen

**Affiliations:** 1 Department of Cell Biology, College of Life Science and Technology, Jinan University, Guangzhou 510632, China; lifu4561@stu2021.jnu.edu.cn (F.L.); minghua_lei01@163.com (M.L.); xiejunyep@163.com (J.X.); gsj2011@jnu.edu.cn (S.G.); liweicai10199@163.com (W.L.); ren_xj06@163.com (X.R.); wangtengccc@163.com (T.W.); 2 National Engineering Research Center of Genetic Medicine, Guangzhou 510632, China; 3 Guangdong Province Key Laboratory of Bioengineering Medicine, Guangzhou 510632, China; 4 Guangdong Provincial Biotechnology Drug & Engineering Technology Research Center, Guangzhou 510632, China; 5 Guangzhou Ocean Land Testing Technology Co., Ltd., Guangzhou 511400, China; linsongxiong@126.com

**Keywords:** cyclopeptide, shark-derived, long-effect, antitumor

## Abstract

Peptides pose a challenge in drug development due to their short half-lives in vivo. In this study, we conducted in vitro degradation experiments on SAIF, which is a shark-derived peptide that we previously studied. The degradation fragments were sequenced and a truncated peptide sequence was identified. The truncated peptide was then cloned and expressed via the *E. coli* system with traceless cloning to form a novel cyclic peptide in vitro oxidation condition via the formation of a disulfide bond between the N- and C-termini, which was named ctSAIF. ctSAIF exhibited high anti-HCC activity and enhanced enzymatic stability in vitro, and retained antitumor activity and good biocompatibility in systemic circulation in a HCC xenograft model. Our study discovered and characterized a novel shark-derived cyclic peptide with antitumor activity, laying a foundation for its further development as an antitumor drug candidate. The study also provided a new solution for peptide drug development.

## 1. Introduction

Liver cancer is one of the six most common cancers in the world, ranking fifth in incidence and fourth in mortality [1]. It is the leading cause of cancer death in Chinese men under the age of 60, which can be divided into primary and secondary categories. Primary liver cancer, also known as hepatocellular carcinoma (HCC), is the most common subtype of liver cancer death, involving multiple molecular mechanisms and stimulation of multiple signaling pathways [2]. Examples include vascular endothelial growth factor, epidermal growth factor receptor (EGFR), insulin growth factor, Ras/extracellular signal-stimulating kinase, mammalian target rapamycin (mTOR), C-mesenchymal epithelial transforming Factor-1 (c-Met), Wnt, and apoptotic signaling. The high mortality rate and poor prognosis of HCC occur because HCC is asymptomatic in its early stages, leading to late diagnosis and significant resistance to conventional chemotherapy and radiotherapy [3,4]. The treatment of hepatocellular carcinoma is based on the Barcelona Liver Cancer (BCLC) staging system, according to the tumor stage and the expected benefit of the main interventions. In principle, surgical resection, liver transplantation, local ablation, and TACE therapy are preferred for patients with early-to-mid-stage hepatocellular carcinoma, while patients at an advanced stage receive mainly drug-based systemic therapy.

The current clinical treatments for HCC are divided between small-molecule chemical drugs, such as lenvatinib and lapatinib, and antibody drugs, such as bevacizumab and atelelizumab [5,6]. Although Sorafenib and regorafenib are recommended first- and second-line therapies for patients with advanced hepatocellular carcinoma (HCC), respectively, both drugs show significant adverse reactions during treatment, such as hypertension and albuminuria in patients treated with lapatinib. Patients treated with sorafenib had more skin reactions and diarrhea on their hands and feet, while antibotics led to an increased risk of bleeding and decreased quality of life scores for both treatments; thus, it is important to develop more effective drugs with fewer side effects [7].

Peptide drugs are compounds with specific therapeutic effects synthesized through chemical synthesis, recombination expression, or extraction from animals and plants. Peptide drugs have unique advantages in the pharmaceutical industry. Compared to small molecule drugs, peptide drugs have higher biological activity and specificity, as well as higher efficiency and safety. Moreover, the metabolites of peptides are amino acids, which have fewer side effects on the human body. Compared to protein drugs, peptide drugs also have many advantages, such as better stability, higher purity, lower production cost, and lower or no immunogenicity [8,9].

Due to their unique advantages, Peptide drugs are widely used in treating diabetes [10], tumors [11,12], hepatitis, and other diseases [13]. For example, in the field of tumor treatment, Liraglutide is a water-soluble nonapeptide that can stimulate the pituitary gland to secrete gonadotropins and is used to treat or relieve various hormone-dependent diseases, such as prostate cancer [14]. In addition, Cilengitide, which is widely used in tumor therapy, exerts its antitumor effects through blocking the binding of integrins to the extracellular matrix, inhibiting integrin-mediated signaling pathways, and, subsequently, blocking cell proliferation, survival, and migration [15,16].

Although peptide drugs have many unique advantages, there are also some shortcomings [17]. They differ from small molecule chemical drugs in terms of chemical structure and physicochemical properties, with the basic unit being amino acid residues and the covalent binding mode being the same as proteins. Therefore, peptides are natural substrates for many proteases [18,19]. Peptide drugs are degraded through the same pathways as endogenous proteins, mainly via the hydrolysis of peptide bonds by proteases in the body. Based on the location of hydrolysis, peptide-degrading enzymes can be classified into endopeptidases and exopeptidases [20,21]. Endopeptidases can break peptide bonds within the peptide drug, while exopeptidases can catalyze the hydrolysis of peptide bonds at the end of the peptide chain but cannot break bonds within the molecule [22,23,24]. Therefore, cyclic peptides are more resistant to proteolytic degradation by proteinases, especially exopeptidases. In addition, the cyclization of peptides also reduces entropy associated with the unfolded state of the peptide, and stabilizes their native folded conformation, thus enhancing their biological activity [25,26,27,28,29]. There are also many naturally occurring cyclic peptides and proteins [30,31,32]. For example, Sunflower Trypsin Inhibitor 1 (SFTI-1) is a bicyclic 14-amino acid peptide found in sunflower seeds that exhibits potent trypsin inhibitory activity [33,34], and other well-known cyclic peptides are insulin, penicillin, cyclosporin, and gramicidin S. Cyclic peptides, which, compared to linear peptides, are proven to show greater potential as therapeutic agents.

Our previous study discovered a novel Shark Angiogenesis Inhibition Factor (SAIF) from shark cartilage, which is composed of 33 amino acid residues and demonstrated potential anticancer effects against breast cancer in vivo [35]. However, due to its unmodified natural peptide structure, SAIF is prone to degradation and aggregation, resulting in low serum stability and pharmacological efficacy. Therefore, optimizing further and modifying its sequence is necessary to obtain a more valuable and promising candidate peptide drug.

Therefore, we first performed in vitro degradation experiments in this study to obtain a truncated peptide sequence with higher stability, which we named tSAIF. Next, through prokaryotic expression [36] and in vitro oxidative cyclization [37,38], a novel cyclic peptide, i.e., ctSAIF, was formed through connecting the N- and C-termini with a disulfide bond. Compared with the unmodified SAIF, ctSAIF showed improved stability, retained high inhibitory activity against several hepatocellular carcinoma cell lines, and demonstrated in vivo antitumor activity in a xenograft model.

## 2. Results

### 2.1. The Analysis and Obtaining of the ctSAIF Peptide Sequence

**Figure 1:**
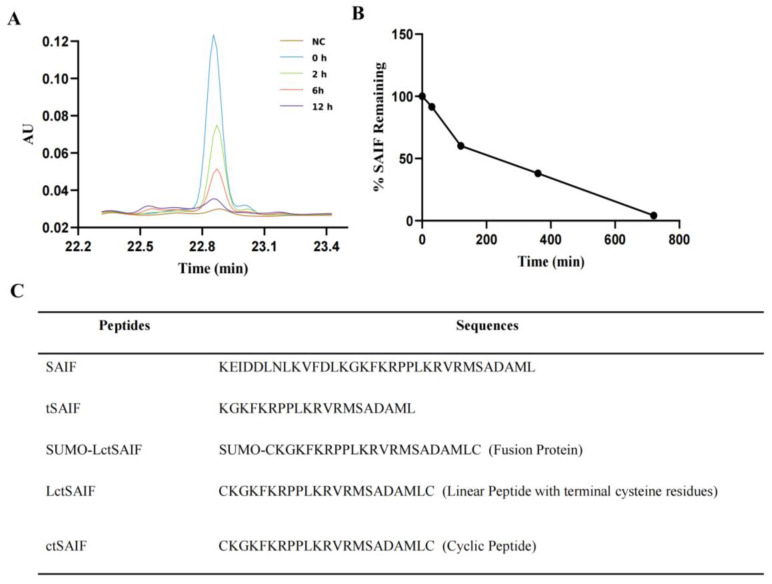
Analysis and acquisition of ctSAIF polypeptide sequence: (**A**) HPLC chromatogram of SAIF serum degradation experiment in vitro; (**B**) Serum degradation curve of SAIF in vitro; (**C**) Amino acid sequence of peptides.

**Figure 2:**
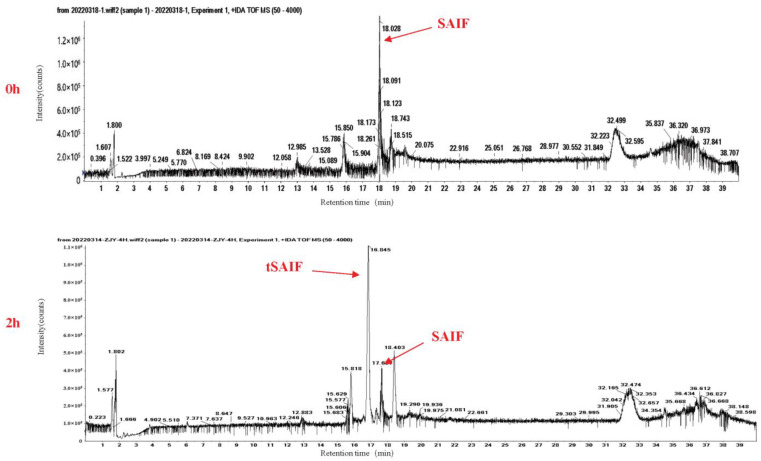
Total ion chromatogram (TIC) of SAIF serum degradation experiment in vitro.

During our investigation into the serum stability of SAIF, we observed poor stability with a short half-life of 2–3 h, as evidenced via HPLC-UV analysis (Figure 1A,B). However, we also observed new peaks with retention time drift (Figure 2), suggesting the presence of degradation products that may exhibit higher stability. Through mass spectrometry analysis, we identified a degradation peak with a molecular weight of 2395.38, which persisted at high levels after incubation with serum for 2 h. Through enumerating all possible degradation peptide sequences from the SAIF sequence using Python software, we identified the corresponding sequence for this degradation peak, which we named tSAIF (Figure 1C). To increase its stability further [39], we expected to perform in vitro cyclization of tSAIF to obtain ctSAIF.

### 2.2. Expression, Purification and Characterization of LctSAIF in E. coli

We obtained the DNA sequence of LctSAIF from its amino acid sequence and synthesized this DNA sequence. We then cloned this DNA sequence into the prokaryotic expression vector pET-21a-His-SUMO, which expresses fusion proteins containing both His and SUMO tags at the N-terminus for easy purification. After induction and purification of the fusion protein LctSAIF-SUMO-His, the tags were removed using SUMO protease to achieve precise expression of the peptide [40].

**Figure 3:**
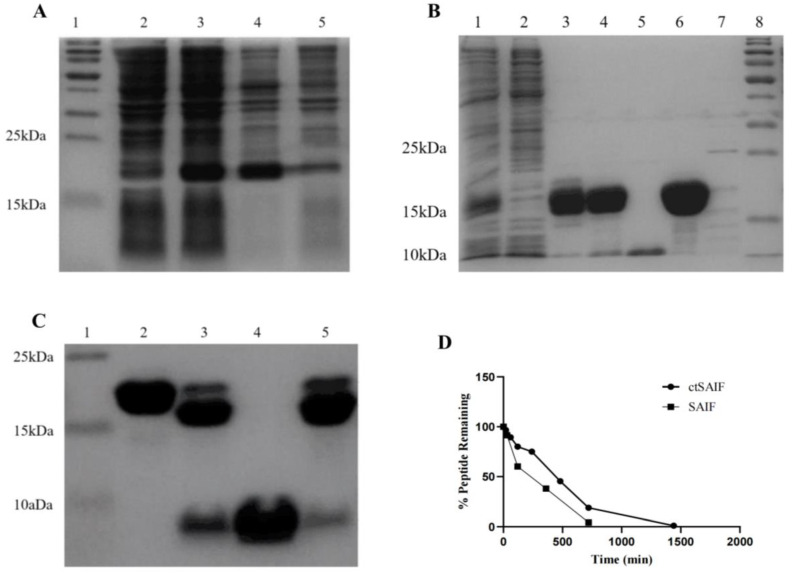
Expression, purification and characterization of LctSAIF in *E. coli*. (**A**) SDS-PAGE results of induction: lane 1: marker; lane 2: pre-induction whole cell lysate; lane 3: post-induction whole cell lysate; lane 4: post-induction cell-free supernatant; lane 5: post-induction cell-free pellet. (**B**) SDS-PAGE results of purification: lane 1: cell lysate; lane 2: Ni-NTA flow-through; lanes 3 and 4: two elution peaks from the Ni-NTA; lane 5: SOURCES 30Q flow-through; lanes 6 and 7: two elu-tion peaks from the SOURCES 30Q; lane 8: marker. (**C**) SDS-PAGE results of purification: lane 1: marker; lane 2: LctSAIF-SUMO; lane 3: Mix after enzymatic cleavage; lane 4: Ni -NTA flow-through; lane 5: Ni-NTA elution peak. (**D**) SAIF and ctSAIF degradation curves in serum in vitro.

As shown in lanes 2 and 3 of Figure 3A, after the transformation of the vector containing LctSAIF into BL21(DE3), induction resulted in abundant expression of a 21 kDa protein compared to the uninduced control. After sonication and centrifugation, most of the induced protein was found in the soluble fraction of the supernatant, as shown in lanes 4 and 5 of Figure 3A.

The bacterial lysate was loaded onto Ni Sepharose 6 Fast Flow, which is a metal chelation affinity chromatography, and the elution was loaded onto ion exchange chromatography Source 30Q, as shown in lane 6 of Figure 3B, resulting in a single band of LctSAIF-SUMO-His with a molecular weight of approximately 21 kDa. After the LctSAIF-SUMO-His was purified, the SUMO-His tag was removed via SUMO enzyme treatment and digested for 16 h at 4 °C. Figure 3C lanes 2 and 3 showed that the LctSAIF-SUMO-His was separated into an 18 kDa SUMO-His and a 3 kDa single-chain LctSAIF. Finally, the enzyme-digested mixture was loaded onto Ni Sepharose 6 Fast Flow to collect the flow-through (Figure 3C lane 4), resulting in a relatively pure single-chain LctSAIF.

To obtain the cyclic peptide, the single-chain LctSAIF was oxidized to form a disulfide bond between the two cysteine residues at the N- and C-termini. Specifically, the peptide solution was treated with 5% DMSO, and the pH was adjusted to 8.5 with NaOH. The mixture was stirred magnetically for 8 h at room temperature, during which Ellman’s reagent was used to monitor free cysteine residues until complete oxidation was achieved [41,42].

**Figure 4:**
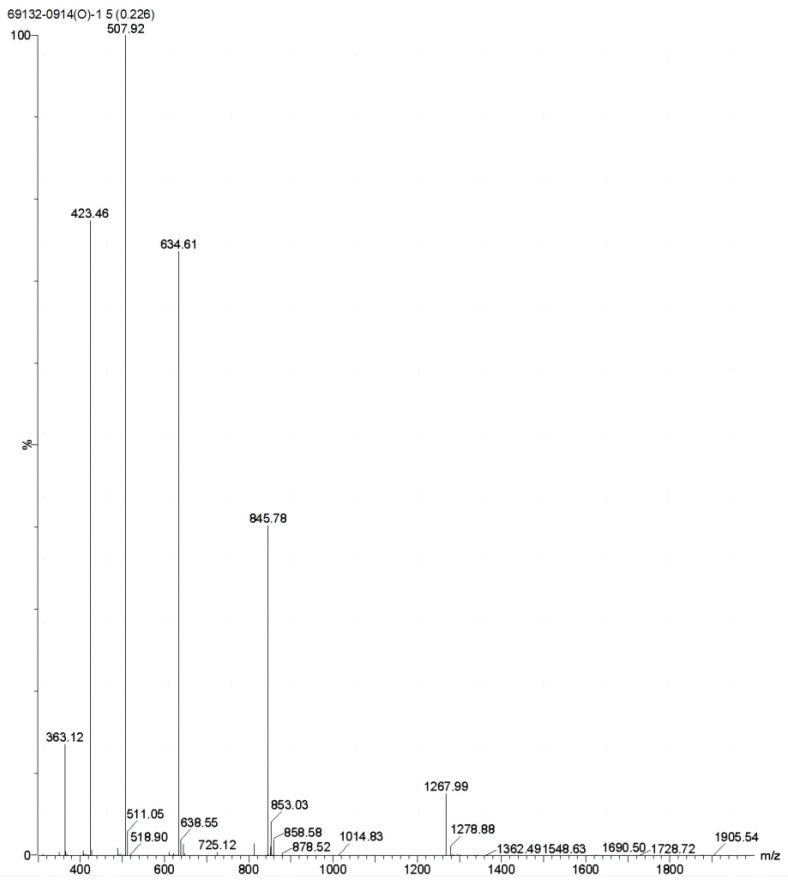
Mass spectrometry results of ctSAIF.

To verify that there was no intermolecular dimer formation of ctSAIF, the oxidized product was analyzed via mass spectrometry, which showed a molecular weight of 2534.20, indicating that no dimer was formed (Figure 4). In summary, we successfully cyclized ctSAIF. Subsequently, a stability experiment was performed on ctSAIF in vitro, revealing that its half-life was extended from approximately 2 h for SAIF to approximately 6 h (Figure 3D).

### 2.3. Inhibition of Hepatocellular Carcinoma Cell Proliferation Using ctSAIF

**Figure 5:**
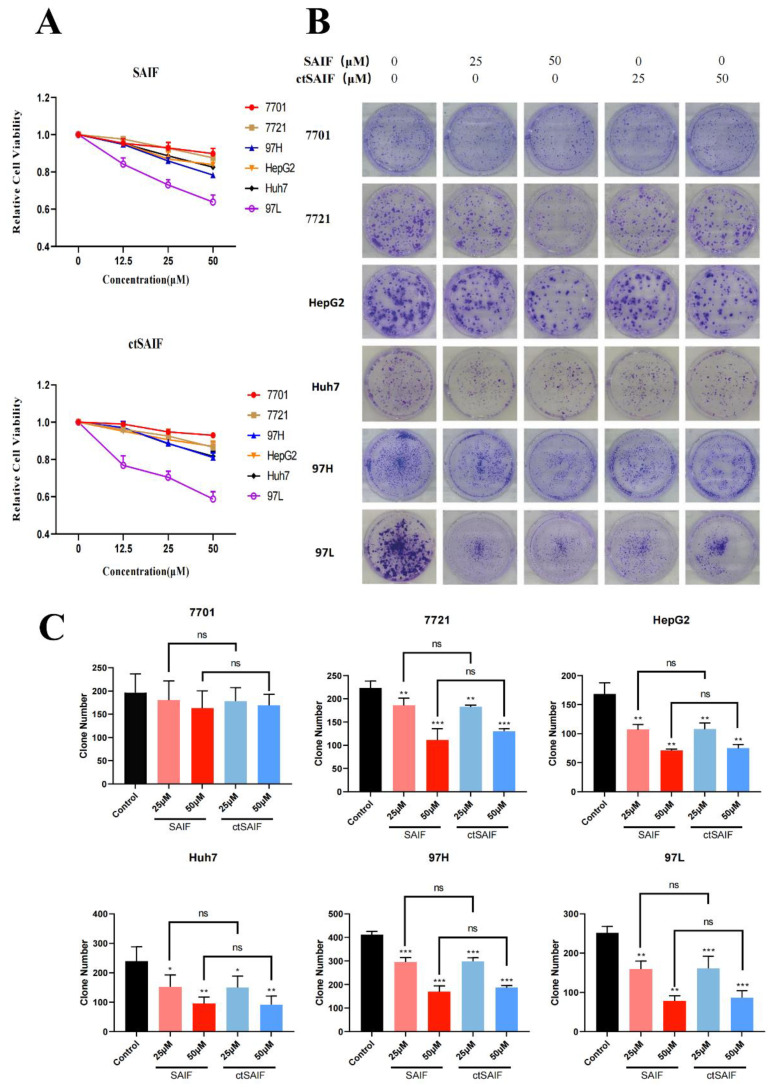
ctSAIF inhibits HCC cell growth: (**A**) Effect of SAIF on the viability of human liver cell line 7701 and hepatocellular carcinoma cell lines (7721, MHCC97H, HepG2, Huh7, MHCC97L); (**B**) inhibitory effect of SAIF on clone formation of human liver cell line 7701 and hepatocellular carcinoma cell lines (7721, MHCC97H, HepG2, Huh7, MHCC97L); (**C**) statistical histogram of cell clone formation. Student’s *t*-test was used to compare values for differences between groups; *p* < 0.05 was considered statistically significant. * represents *p* < 0.05, ** represents *p* < 0.01, *** represents *p* < 0.001 and ns represents *p* > 0.05.

To determine whether ctSAIF possesses the same antitumor activity as SAIF, we analyzed its effects on liver cancer cells using a human hepatocellular carcinoma (HCC) cell model. Firstly, cell proliferation assays were performed to evaluate the inhibitory effects of SAIF and ctSAIF on five HCC cell lines (7721, HepG2, Huh7, MHCC97H, and MHCC97L). The results showed that both SAIF and ctSAIF significantly reduced cell viability in a concentration-dependent manner after treatment for 48 h (Figure 5A). Additionally, the colony formation assay (Figure 5B,C) demonstrated that SAIF and ctSAIF had long-term inhibitory effects on HCC cell proliferation. Furthermore, the toxicity of SAIF and ctSAIF on normal human liver cell line 7701 was evaluated, and the results showed no significant toxicity for both peptides.

### 2.4. Inhibition of Liver Cancer Transplant Growth Using ctSAIF

**Figure 6:**
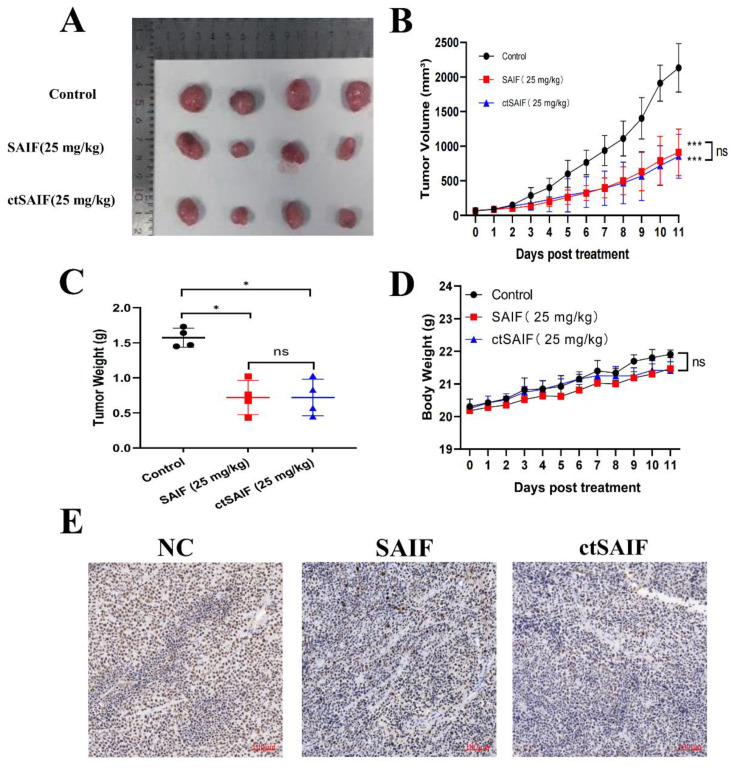
ctSAIF inhibits growth of MHCC97L xenografts: (**A**) tumor size visual graph; (**B**) tumor volume curve; (**C**) tumor weight after 14 days of SAIF treatment; (**D**) body weight changes in nude mice during drug treatment; (**E**) Ki67 staining of tumor sections in nude mice. Student’s *t*-test was used to compare values for differences between groups; *p* < 0.05 was considered statistically significant. * represents *p* < 0.05, *** represents *p* < 0.001 and ns represents *p* > 0.05.

To better study the in vivo antitumor effects of SAIF and ctSAIF, we selected the most sensitive cell line MHCC97L in the HCC cell lines for the subcutaneous transplantation model in nude mice. The weight and tumor growth of nude mice were monitored every two days for a total of 14 days of treatment. The results showed that compared to the model group, both the SAIF treatment group and the ctSAIF treatment group significantly inhibited the growth of MHCC97L transplant tumors, and the average tumor volume (Figure 6A,B) and average tumor weight (Figure 6C) were significantly reduced. During the treatment process, there were no significant changes in the weight of nude mice in each group (Figure 6D). Furthermore, Ki67 staining of tumor tissue further confirmed that both SAIF and ctSAIF could inhibit the growth of tumor cells (Figure 6E).

## 3. Discussion

Based on the proven antitumor activity of the polypeptide SAIF, we obtained a novel cyclic peptide, i.e., ctSAIF, through acquiring its degraded peptide and cyclization. The results showed that ctSAIF retained the antitumor activity of the original SAIF and improved the resistance to enzymatic degradation. The obtaining of this cyclic peptide provides a solid material foundation for the next step in drug development.

In this study, we provided a strategy and pathway for optimizing existing peptides to achieve long-lasting effects. We screened and optimized peptides from fragments produced via proteolytic degradation in plasma. We first obtained various degradation fragments of SAIF through in vitro serum degradation, and then used LC-MS detection and Python-assisted analysis to identify the shortest peptide sequence with the longest half-life. Linear peptides were obtained via recombinant expression, followed by in vitro cyclization to produce the cyclic peptide ctSAIF. A comparison between the stability of SAIF and ctSAIF showed that their half-lives increased from approximately 2 h for the original SAIF to about 6 h for ctSAIF. Both in vivo and in vitro experiments confirmed that ctSAIF still possessed the in vitro antitumor activity of the original SAIF, indicating that ctSAIF was successfully screened and optimized as a new and longer-lasting candidate for cancer treatment.

Furthermore, the improved stability of ctSAIF initially demonstrated the feasibility of using cyclization to block the terminal of a peptide, which works through inhibiting the recognition and degradation of terminal amino acid residues using exopeptidase. However, for some endopeptidases that recognize non-terminal amino acids, further modification may be needed to improve stability, such as the introduction of non-natural amino acids, and modifications to both ends of the peptide, such as terminal fatty chain modification, can enhance the structural stability of polypeptides due to the increased hydrophobic effect.

This study did not investigate the mechanisms through which ctSAIF inhibits tumor growth in depth. However, in future research and development, relevant studies on the homologous protein Tn1 should be referenced for comparative analysis [43,44,45,46]. It was reported that Tn1 may have two possible mechanisms for inhibiting tumor growth. Firstly, as an inhibitor of calcium channel regulatory protein, it exerts negative feedback and promotes the overexpression of calcium-regulating proteins, leading to the inhibition of the proliferation and migration of smooth muscle cells [47]. Secondly, as an inhibitor of angiogenesis [48,49], it exerts antitumor effects through inhibiting the VEGF/VEGFR pathway [50,51,52]. Structural analysis of ctSAIF indicates that it is rich in positively charged arginine residues with a pI of 10, while the pI of VEGF is 8.5; both are positively charged under physiological conditions and can easily bind to heparin [53,54,55]. Therefore, we predict that ctSAIF may inhibit tumor angiogenesis through competitively binding to VEGFR with VEGF [56].

In summary, this study screened and demonstrated that ctSAIF is a potentially safe and effective new cyclic peptide for tumor inhibition. Exploring the specific mechanisms of ctSAIF to fully realize its drug potential is undoubtedly our future research direction.

## 4. Materials and Methods

### 4.1. Cell Lines

Normal human liver cells QSG-7701 and Hepatocellular Carcinoma Cell lines 7721, Huh7, MHCC97H, MHCC97L, and HepG2 were all obtained from the Institute of Biomedical Sciences, Jinan University. All cells were cultured in DMEM medium (Gibco, Grand Island, NE, USA) supplemented with 10% fetal bovine serum (Gibco, Grand Island, NE, USA). The cell lines were incubated in a humidified atmosphere with 5% CO_2_ at 37 °C.

### 4.2. Materials

Seamless Cloning Kit and SUMO protease were purchased from Beyotime Biotechnology (Shanghai, China). The expression vector pET-21a-His-SUMO and *Escherichia coli* strain BL21(DE3) were stored by Institute of Biomedicine, Jinan University, while primers were synthesized by Sangon Biotech (Shanghai, China). Ni-Sepharose 6 Fast Flow column was purchased from GE Healthcare (Marlborough, MA, USA). Isopropyl-1-thio-β-galactopyranoside was purchased from Genebase (Guangzhou, China). Restriction endonuclease and Agarose Gel Extraction kits were purchased from TAKARA Bio. Plasmid extraction kits were purchased from TIANGEN (Beijing, China). Other chemical reagents were purchased from Sigma-Aldrich (Saint Louis, MO, USA), were reagent grade, and could used without further purification.

### 4.3. Cell Proliferation Assay (CCK-8 Assay)

Cells in the logarithmic growth phase with good viability were seeded into 96-well plates at a density of 2500 cells per well and incubated for 24 h at 37 °C with 5% CO_2_. The old medium was removed, and the cells were starved with DMEM medium containing 0.5% FBS for 24 h. Peptides were diluted with 0.5% starved medium to different concentrations and added to each well with the same volume. The cells were then incubated for an additional 48 h at 37 °C. After removing the medium, 100 μL of CCK-8 working solution was added to each well, and the plate was incubated for 1 h at 37 °C with 5% CO_2_. Finally, the absorbance at 450 nm of each well was measured using a multifunctional microplate reader. Cell viability (%) was equal to (A_sample_ − A_blank_)/(A_control_ − A_blank_) × 100%. All experiments were performed with four duplicates.

### 4.4. Cell Colony Formation Assay

Cells in the logarithmic growth phase were seeded at a density of 1000 cells per well in 6-well plates and cultured for 24 h. After removing the old medium, 2 mL of the prepared peptide solution at different concentrations was added, and the control group was complemented with 2 mL of the complete medium. After continuous treatment for 14 days, the medium was discarded, and the cells were gently washed 2–3 times with PBS buffer. Next, 500 μL of 4% paraformaldehyde was added to each well, and the cells were incubated for 30 min, followed by removal of the paraformaldehyde and air-drying. A total of 1 mL of 0.1% crystal violet solution was added to each well and incubated for 30 min, followed by gentle washing 3 times with PBS buffer. After air-drying the plate, the colonies were photographed and counted.

### 4.5. In Vitro Serum Stability Assay of Peptides

The serum obtained from healthy individuals was centrifuged at a low speed and mixed with peptides at a ratio of 9:1 to achieve a peptide concentration of 1 mg/mL. The peptide–serum mixture was then incubated at 37 °C for different periods. After reaching the pre-determined time point (0, 10, 20, 30, 60, 120, 240, 480, 960, or 1440 min), pure trifluoroacetic acid was added to each sample to reach a final concentration of 10%. The mixture was then vortexed and centrifuged for 10 min at 13,000 rpm, and the supernatant (10 μL) was collected and stored in HPLC vials for analysis.

HPLC-UV analysis was performed using a C18 analytical column (Thermo Acclaim300 C18 LC ColuMns, 4.6 mm diameter, 150 mm length, Catalog #: 060266) and a Waters ACQUITY Arc Bio instrument, following the method shown in Table 1. The target peak was determined based on the retention time of pure peptides, and on this basis, detect peptides were degraded at different times and their peak area recorded to generate a degradation curve. The degradation rate of peptides was equal to (Area_sample_ − Area_blank_)/(Area_control_ − Area_blank_) × 100%. The area of the blank was equal to the peak area detected after mixing peptides with ultrapure water at the same initial concentration. The signal-to-noise ratio of all collected data was greater than 10.

### 4.6. Mass Spectrometry Analysis

According to the sample preparation and chromatography methods in Table 1, we replaced trifluoroacetic acid (TFA) with formic acid and performed an analysis using liquid chromatography-mass spectrometry (LC-MS) (AB SCIEX 500R, TOF MS). We then obtained the total ion chromatogram (TIC) and the mass spectrum and calculated the molecular weight of the main peak based on the mass-to-charge ratio for quantitative analysis.

### 4.7. Construction of Recombinant Expression Vector

The DNA sequence was obtained according to the conversion of the amino acid sequence of LctSAIF, which was modified to accommodate *Escherichia coli*’s codon preference, while the amino acid sequence of the peptide remained unchanged. Chemical synthesis of the DNA sequence fragment corresponded to ctSAIF (GTGAGCAGATTGGAGTGCAAGGGCAAGTTCAAGAGGCCCCCCCTGAAGAGGGTGAGGATGAGCGCCGACGCCATGCTGTGCGATCCGAATTCGAGCT), which contained homologous arms of pET-21a-His-SUMO at both ends. The vector pET-21a-His-SUMO was digested with the restriction endonuclease *Bam*H Ⅰ (TAKARA). Digestive products were subjected to 1% agarose gel electrophoresis, extracted products from agarose gel, and purified using TaKaRa MiniBEST Agarose Gel DNA Extraction Kit Ver.4.0. The linearized vector was then mixed with the ctSAIF gene containing homologous arms and enzyme mixture. The homologous recombination reaction was carried out for 15 min at 50 °C [57], which resulted in the cloning of the ctSAIF gene into the vector pET-21a-His-SUMO. The SUMO fusion tag was added to improve the solubility and expression of the fusion protein [58], and the His tag allowed purification v Ni-NTA affinity chromatography and identification via Western blot with an anti-6 ×His antibody. The cloned products were transformed into clonal bacteria DH 5α, uniformly spread onto an agar plate containing Amp, and cultured overnight in an inverted incubator. Single colonies were selected, amplified, and extracted via the plasmid according to the kit instructions. Finally, the extracted plasmid was verified via sequencing.

### 4.8. Expression and Purification of Cyclic Peptide ctSAIF

After sequence verification, the recombinant plasmids were transformed into expression strain BL21 (DE3). The bacteria containing the recombinant plasmids were grown in fresh LB medium on a shaking incubator until the OD600 reached 0.6–0.8 at 37 °C. Next, 2 mL of the culture was taken as a negative control for subsequent experiments, and the remaining culture was induced with a final concentration of 1 mM IPTG (Isopropyl β-D-Thiogalactoside) for another 4–5 h in 37 °C. After induction, the bacterial cells were collected via centrifugation for 30 min at 4000 rpm, and the soluble expression of the fusion protein was confirmed via SDS-PAGE. The cells were then sonicated, and the supernatant was collected and purified via Ni Sepharose 6 Fast Flow and SOURCE 30Q. The fusion protein LctSAIF-SUMO-His was obtained and treated with SUMO protease (SUMO protease:fusion protein = 1 IU:2 μg) at for 16 h 4 °C, and specifically recognized SUMO-modified or SUMO domain-containing proteins and cleaved the peptide bond following the Gly-Gly motif at the carboxyl-terminal (C-terminal) of x-Gly-Gly-x peptide sequences. This enzymatic activity enabled the removal of SUMO modifications or the SUMO domain from SUMO-fused expressed proteins. The cleaved SUMO-His tag was then removed via Ni Sepharose 6 Fast Flow to obtain the purified ctSAIF (purity of SDS-PAGE > 95%). All the purified peptide was freeze-dried and stored at −20 °C. The molecular weight of ctSAIF was analyzed via mass spectrometry. The BCA method was used for all protein quantification, which can avoid the interference of the solution during purification.

### 4.9. Xenograft Model

All animal experiments were performed in compliance with the ethical requirements of the Experimental Animal Center of Jinan University (Ethics Approval Number: 20220612-01). Male BALB/c-nude mice were purchased from Guangdong GemPharmatech Co., Ltd. These mice were fed with food and purified water at room temperature in the SPF laboratory. MHCC97L cells (0.1 mL of physiological saline containing 1 × 10^7^ cells) were subcutaneously injected into the armpit of 5-week-old male BALB/c-nude mice. Tumor volume and weight were measured every 2 days, and tumor volume was calculated using the following formula: (short axis)^2^ × (long axis)/2. When the tumor volume reached approximately 50 mm^3^, these mice were weighed and randomly divided into 3 groups (n = 4): the model group, the SAIF group (25 mg/kg), and the ctSAIF group (25 mg/kg). SAIF and ctSAIF were administered via tail vein injection at a dose of 25 mg/kg. The peptide was dissolved in saline solution to 5 mg/mL, and the dosage volume of each mouse was calculated using the following formula: (25 mg/kg × body weight)/(5 mg/mL). After 14 days of treatment, the mice were sacrificed, and the tumors were collected for histopathological examination.

### 4.10. Histopathological Examination

The tumors were excised, fixed in a 10% paraformaldehyde solution, embedded in paraffin, and cut into 5 mm-thick slices for staining. Slides were stained with rabbit monoclonal antibody to Ki67 (Ventana Medical System Inc., Tucson, AZ, USA) to observe the proliferating cells, and the slides were viewed on a Nikon Eclipse E600 microscope (Nikon Corporation, Tokyo, Japan).

### 4.11. Statistical Analysis

Statistical differences were determined using the one-way analysis of variance test of Student’s *t*-test. Data were expressed as mean and standard deviation. A value of *p* < 0.05 was considered statistically significant.

## Figures and Tables

**Table 1:** HPLC method for detecting peptides.

Time (min)	Solvent A (0.1%TFA in Double-Distilled Water)	Solvent B (0.1%TFA in Acetonitrile)
0	95%	5%
27	75%	25%
28	5%	95%
33	5%	95%
40	95%	5%
45	95%	5%

## Data Availability

Not applicable.
